# Letter to the editor concerning “Probing artificial intelligence in neurosurgical training: ChatGPT takes a neurosurgical residents written exam” by Bartoli, Aet al.

**DOI:** 10.1016/j.bas.2024.102918

**Published:** 2024-08-08

**Authors:** Vanitha Marunganathan, Ajay Guru

**Affiliations:** Department of Cariology, Saveetha Dental College and Hospital, Saveetha Institute of Medical and Technical Sciences, Saveetha University, Chennai, 600077, Tamil Nadu, India

Dear Editor,

The study by Bartoli et al. (2024) entitled “Probing artificial intelligence in neurosurgical training: ChatGPT takes a neurosurgical residents written exam” on the use of ChatGPT in neurosurgical education presents a novel approach to integrating artificial intelligence into medical training. This research explores the capabilities of ChatGPT in generating and answering questions similar to those found in a neurosurgery residents' exam. Furthermore, the inclusion of both open-ended and multiple-choice questions offers a comprehensive evaluation of ChatGPT's proficiency in addressing different types of medical queries. Despite these strengths; the study has several notable setbacks. Firstly, the small sample size of only 10 residents and the AI model limits the generalizability of the findings. A larger participant pool would yield more robust and reliable data. Secondly, the study's scope was limited as only 4 out of 50 questions were generated by ChatGPT, excluding questions involving images or institutional protocols. This narrow focus may not fully capture the potential or limitations of ChatGPT in generating relevant medical questions. Additionally, the iterative process required for question generation indicates that the initial outputs from ChatGPT were not always precise or relevant, raising concerns about the practical usability of the AI without significant human oversight. Moreover, the incident where ChatGPT provided a gynecological answer to a neurosurgical question underscores the critical issue of context and domain-specific knowledge, which could severely limit its application in high-stakes medical education.

To enhance the robustness and applicability of the study, several improvements are suggested. Increasing the sample size and diversity of participants would provide more comprehensive insights. Broadening the scope of AI-generated content to include more questions and various types of queries, such as those involving images and specific institutional protocols, could better demonstrate ChatGPT's capabilities. Introducing more detailed evaluation metrics beyond simple scoring, such as analyzing the reasoning behind incorrect answers or partial credits, would offer deeper insights into the AI's strengths and weaknesses. Future studies could also explore hybrid models where AI-generated questions are reviewed and refined by human experts, potentially enhancing the quality and relevance of the questions (Sudhakaran, 2024). Moreover, developing domain-specific models or fine-tuning existing models like ChatGPT on neurosurgical data could improve accuracy and relevance, particularly in highly specialized fields.

For future research, it is recommended to conduct longitudinal studies to examine the long-term impact of AI in medical education, including potential changes in learning outcomes and resident performance. Comparing ChatGPT with other AI tools or models specialized in medical education could also highlight relative strengths and weaknesses. A more comprehensive and methodologically rigorous approach could yield deeper insights and more practical applications for AI in neurosurgical training. By addressing these limitations and exploring suggested improvements, future research can better harness the potential of AI to enhance medical education and training.

## Declaration of competing interest

The authors declare that they have no known competing financial interests or personal relationships that could have appeared to influence the work reported in this paper.
